# Microsaccadic modulation in goal-directed reaching

**DOI:** 10.1167/jov.26.3.4

**Published:** 2026-03-13

**Authors:** Jolande Fooken, Renato Moraes, Allison M. Scott, J. Randall Flanagan

**Affiliations:** 1 Department of Psychology and Centre for Cognitive Science, Technical University of Darmstadt, Darmstadt, Germany; 2 Department of Psychology and Centre for Neuroscience Studies, Queen's University, Kingston, Ontario, Canada; 3 School of Physical Education and Sport of Ribeirão Preto, University of São Paulo, Ribeirão Preto, São Paulo, Brazil; 4 Schulich School of Medicine and Dentistry, Western University, London, Ontario, Canada

**Keywords:** reaching, microsaccades, sensorimotor attention, event prediction

## Abstract

Previous work has demonstrated that microsaccades play a key role in both preparation for upcoming sensory stimuli and visual attention. Microsaccadic modulation prior to a predictable event is observed with visual, tactile, and auditory stimuli, suggesting a common function across sensory modalities. This study investigated how microsaccade modulation relates to sensorimotor control in goal-directed reaching. We designed a task in which participants positioned their hand (represented as a cursor) at a start position and then fixated a target, which changed color after a variable delay. Depending on the condition, in response to this color change (go cue), participants waited for the trial end (cue monitoring), watched a cursor move with biological motion from the hand start position to the target (cursor tracking), or reached to the target either with (reach visible) or without (reach invisible) vision of the hand. We found that the rate of microsaccades was consistently reduced in anticipation of the visual cue. Microsaccade reduction immediately before the go cue was greatest in conditions that required active hand movement, suggesting that movement preparation added attentional load to the sensorimotor system. Following the go cue, microsaccades remained reduced in all conditions, indicating that not only external events but also self-generated movements are associated with event prediction. Yet, the reduction in microsaccade rate was not greater in the movement conditions, suggesting that sensorimotor control processes that are involved in predicting the sensory consequences of motor commands neither enhance nor reduce microsaccade rate.

## Introduction

Eye movements can be viewed as indicators of perceptual and cognitive processes (König et al., 2016; Schütz, Braun, & Gegenfurtner, 2011; Spering, 2022; Yarbus, 1967). In the words of Eileen Kowler, eye movements “are a response to a representation of the visual world. And, not just a representation of the objects or the visual scene, but also information about plans, goals, interests, and probable sources of rewards or useful information. Even expectations about future events.” (Kowler, 2011, p. 1457). Thus, eye movements are not only sensitive to current visual input but are also indicative of observers’ expectations of future events, including events arising as a consequence of planning actions (Fooken & Spering, 2019; Hayhoe, McKinney, Chajka, & Pelz, 2012; Kowler, 1989).

Predicting relevant events in the environment not only is important for the control of eye movements itself but can more generally aid the coordination of perceptual, cognitive, and motor processes (Fiehler, Brenner, & Spering, 2019; Fooken et al., 2023). Humans can quickly learn and exploit the temporal structure of events that occur in their environment to proactively prepare for incoming sensory information and upcoming motor action (for a review, see Nobre & van Ede, 2018). In recent years, microsaccades, small movements (<1 degree of visual angle) of the eyes that occur during fixation, have been shown to be a behavioral marker of higher-level attentional processes (Engbert & Kliegl, 2003; Gu et al., 2024; Hafed & Clark, 2002; Kowler, 2024). It has been shown that microsaccade rates in human observers are systematically reduced in anticipation of expected visual (Amit, Abeles, Carrasco, & Yuval-Greenberg, 2019), auditory (Abeles, Amit, Tal-Perry, Carrasco, & Yuval-Greenberg, 2020), and tactile (Badde, Myers, Yuval-Greenberg, & Carrasco, 2020) events.

Whereas previous work has investigated changes in microsaccade control in anticipation of externally generated sensory events, in real-world tasks predictable events may also be linked to ongoing movement control. For example, when grasping an object, the times at which the object is first contacted by the digits and then lifted off the surface are predicted by the actor, and temporal mismatches between predicted and actual sensory events can trigger task-protective corrective actions (Johansson & Flanagan, 2009). In goal-directed reaching, eye and hand movements are coordinated in stereotypical ways (for a review, see de Brouwer, Flanagan, & Spering, 2021), such that the eyes fixate the target object until around the time that the hand arrives (Ballard, Hayhoe, Li, & Whitehead, 1992; Epelboim et al., 1995; Johansson, Westling, Bäckström, & Flanagan, 2001; Neggers & Bekkering, 2000; Neggers & Bekkering, 2001). Fixating the reach target supports hand movement control by providing peripheral vision of the hand- and gaze-related signals to rapidly correct movement errors (de Brouwer, Gallivan, & Flanagan, 2018; Dimitriou, Wolpert, & Franklin, 2013; Paillard, 1996; Saunders & Knill, 2003; Saunders & Knill, 2004). When the hand is in the vicinity of the reach target, central vision of the target and hand can guide contact and check goal completion (Land & Hayhoe, 2001; Land, Mennie, & Rusted, 1999; Säfström, Johansson, & Flanagan, 2014).

**Figure 1.**
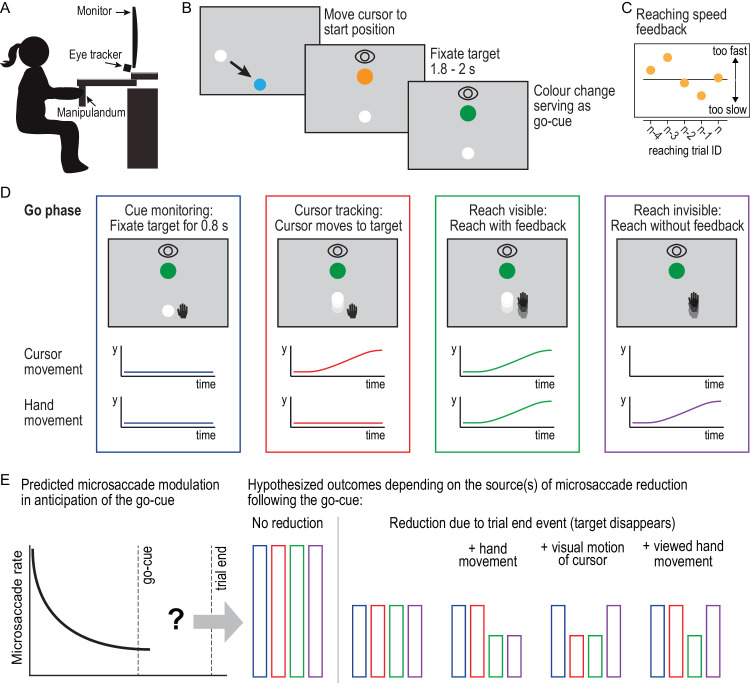
Experimental paradigm and expected modulation of microsaccade rates. (**A**) Participants performed reaching movements by moving the handle of a robotic manipulandum in the horizontal plane while viewing visual targets on a vertical screen. (**B**) At the start of each trial, participants moved the white cursor, representing the right-hand position, to the start position. Participants were instructed to fixate the orange fixation target. When the target changed color, participants performed one of four conditions. (**C**) In trials that required reaching movements, participants received movement speed feedback. (**D**) In the go phase, participants always maintained fixation at the fixation target. In cue monitoring, the fixation target disappeared after 0.8 second. In cursor tracking, the cursor (but not the hand) moved from the start position to the fixation target. In the reach visible condition, participants reached from the start position to the fixation target with the cursor being visible. In the reach invisible condition, participants reached from the start position to the fixation target without visual cursor feedback. (**E**) Hypothesized reduction of the microsaccade rate during the movement phase depending on the sensorimotor processes related to modulation.

In the current study, we investigated the relationship between microsaccade modulation and sensorimotor control in goal-directed reaching. Human participants used a robotic manipulandum to perform goal-directed reaching movements that they viewed on a computer monitor (Figure 1A). In each trial, the participant first moved a cursor, representing their hand position, to a fixed start location and then fixated a visual target that was also a reach target in some conditions. While the participant fixated the target, we presented a predictable sensory event—a color change of the target—that served as a go cue (Figure 1B). In the two reaching conditions, the participant was asked to reach, at a consistent movement speed across trials, to the fixated target following the go cue (Figure 1C). In the reach visible condition, the cursor remained visible throughout the trial, whereas in the reach invisible condition, vision of the cursor was removed when the cursor reached the start position. In two control conditions, participants did not move their hand and either saw a natural cursor movement (i.e., with biological motion) from the start location to the target (cursor tracking) or waited until the trial ended (cue monitoring) (Figure 1D).

We hypothesized that the rate of microsaccades that participants elicited while fixating the target would gradually decrease in anticipation of the go cue (Figure 1E, left panel). Following the go cue, we tested several alternative hypotheses of how microsaccade rates would compare between conditions (Figure 1E). One possibility is that the reduction in microsaccade rate could be exclusively linked to the expectation of the go cue and not be affected by events following this cue. In this case, we would expect that microsaccade rates would increase to their initial level after the go cue in all conditions. A second possibility is that the reduction in microsaccade rate would be observed in anticipation of one or more secondary sensory events marking the end of either the movement or the trial. This could be a visual or proprioceptive event linked to the arrival of the hand at the target in the two movement conditions, a visual event linked to the arrival of the cursor at the target (in the cursor tracking condition), or the disappearance of the target at the end of the trial (in all conditions). In this case, the pattern of microsaccade modulation would be the same in all conditions. Third, the reduction in microsaccade rate following the go cue could be linked to the predictability of target attainment associated with active movement control. In this case, we would expect the two movement conditions (move visible and move invisible) to be characterized by a greater level of microsaccade reduction compared with the other conditions. Fourth, the reduction in microsaccade rate following the go cue could be linked to the predictability of the visual motion of the cursor from the start location to the target. In this case, we would expect the rate of microsaccades to be more reduced in the cursor monitoring and reach visible conditions compared with the other conditions. Finally, a greater level of reduction might only occur in the condition where both the visual and the sensorimotor signals are predictive of target attainment (reach visible).

## Methods

### Participants

Seventeen individuals (four males, 13 females; ages 18–20 years, mean =18.9 years) participated in the experiment. Participants were recruited from the undergraduate student population at Queen's University through the Department of Psychology Participant Pool. All participants were compensated with one psychology course credit. Before the experiment, participants read a letter of information and signed an informed consent form. Upon completion of the experiment, participants were debriefed. All participants were required to have normal or corrected-to-normal vision and no history of neurological conditions. This study was approved by the Queen's General Research Ethics Board.

### Apparatus

Participants were seated in an adjustable chair with their head stabilized on a chin and forehead rest mounted in front of a vertical computer monitor (70 × 40 cm, 1920 × 1080 pixels) that presented the visual stimuli and a monocular video-based eye tracker that recorded eye position at 500 Hz (EyeLink 1000; SR Research, Kanata, ON, Canada). Participants performed right-hand reaching movements in the horizontal plane by grasping and moving the handle of a planar robotic manipulandum (Kinarm End-Point; BKIN Technologies, Kingston, ON, Canada) (Figure 1A). This device recorded the position of the hand (i.e., handle) at 1000 Hz, which was shown as a circular cursor on the vertical monitor. Note that the mapping between hand and cursor movements was like the mapping of a standard mouse, with outward and rightward hand movements in a horizontal plane resulting in upward and rightward cursor movements on the vertical monitor of the computer. In addition, there was a 1:1 correspondence between the hand position and the cursor position. Thus, a 20-cm hand movement in the horizontal plane resulted in a 20-cm cursor movement on the monitor. Because we were measuring both eye and hand movements, we decided to report all movement-related values in centimeters. In our setup, 1 cm corresponded to 1.5 visual degrees (viewing distance = 37 cm).

### Condition and stimulus

The hand start position, fixation target, and cursor (when visible) were presented on the monitor (Figure 1B). In all conditions, the cursor (white, 1-cm diameter) was visible at the start of the trials, and the participants began each trial by using the manipulandum to move the cursor to the start position, marked by a blue circle (2-cm diameter) located 7 cm from the bottom of the screen. When the participant was in position, an orange circle (2-cm diameter) appeared 20 cm vertically above the blue circle. Participants fixated on this stimulus for a delay period between 1.8 and 2 seconds. This delay period aligns with microsaccadic research, in which the greatest effects of microsaccade modulation are found within a 2-second delay prior to stimulus presentation (Abeles et al., 2020). If fixation was lost during the delay period, a “fixation” message would appear on the screen, and the delay period restarted. Following the delay period, the orange target circle turned green, acting as a go signal (Figure 1B). Following the go signal, the task followed condition-specific events (Figure 1D):

• *Cue* *monitoring*—After the go signal (change in target color from orange to green) and a 0.3-second delay (to account for reaction time), participants maintained fixation on the target until it disappeared 0.8 second later.
• *Cursor* *tracking*—Following the go signals and a 0.3-second delay, participants used peripheral vision to monitor a cursor moving from the start position to the target, on which the eyes remained fixated. The vertical position (y) of the cursor moved following a minimum jerk trajectory with a randomly selected movement duration between 1.15 and 1.35 seconds, simulating a biological reach movement (Flash & Hogan, 1985). Specifically, for given movement duration (d) and amplitude (a), the vertical position as a function of time, y(t), is defined as follows:
  $$\begin{array}{c} y\left(t\right)=a\left(10{\left(\frac{t}{d}\right)}^{3}+15{\left(\frac{t}{d}\right)}^{4}+6{\left(\frac{t}{d}\right)}^{5}\right) \end{array}$$
  To further increase the naturalness of the cursor movement, the cursor movement to the target was slightly curved, bowing out to the left or right (randomly selected), to a maximum of 0.25 cm at the midpoint of the movement, before returning to midline at the target. The trial was complete when the cursor reached the target.
• *Reach* *visible*—When the go signal appeared, participants reached straight ahead with their hand to the target. The cursor remained visible throughout the reach movement. The trial was complete when the cursor was inside the target area for 500 ms.
• *Reach* *invisible*—When the go signal appeared, participants reached their hand to the target; however, no visual feedback movement was provided (i.e., the participants could not see their cursor throughout the reach movement). The trial ended when the hand velocity was below the threshold of 10 cm/s for a duration of 200 ms.

After each trial, participants received feedback regarding their average hand velocity between 25% and 75% of the *y* distance to the target. Specifically, they viewed a rectangular box with a central horizontal line that represented the goal average velocity of 20 cm/s. The top and bottom of the box represented average speeds of between 40 and 0 cm/s. Participants viewed the average speeds of their last five trials.

### Procedures

The eye tracker was calibrated at the start of each block. Participants then completed all four conditions, with 60 trials per condition (240 total). Before each condition, participants received verbal condition-specific instructions. All participants began the experiment by completing the cue monitoring condition, followed by the cursor tracking condition. We started with the cue monitoring condition, as this served as a baseline condition that required the least mental load. Moreover, we wanted to ensure that participants were familiar with the task setup before performing the active reaching conditions. The order of the other two movement conditions was randomized. Following the completion of each condition, a rest break was given to minimize fatigue and sustained fixation effects (Di Stasi et al., 2013; Poletti & Rucci, 2016). Despite our efforts to reduce mental fatigue, we cannot rule out that the movement conditions were more strongly affected by fatigue than cue monitoring and cursor tracking. The experiment lasted approximately 60 minutes. Upon completion of all conditions, participants were compensated and debriefed accordingly.

### Eye and hand movement analysis

**Figure 2.**
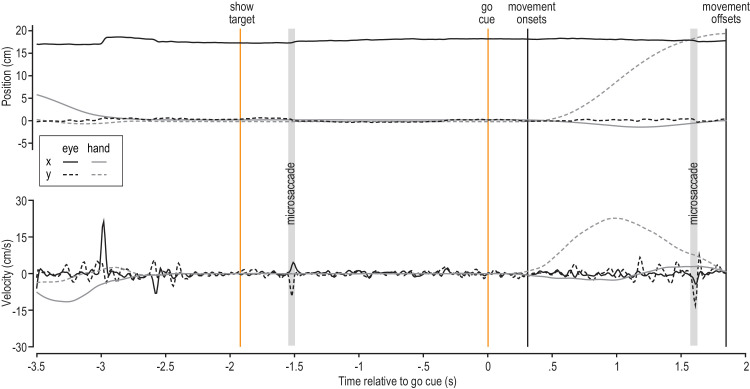
Illustrative hand and gaze position (top) and velocity (bottom) across a single trial. In this reach-visible trial, the participant fixated the reach target throughout the trial (black lines) and moved the hand from the start location (0,0) to the reach goal (0,20; gray lines). Relevant events are indicated by vertical lines, and microsaccades are highlighted by gray-shaded areas.

Figure 2 illustrates the change in *x*,*y* hand and eye position (top) and *x*,*y* hand and eye velocity (bottom) across a single example trial in the reach visible condition. Initially, the participant moved the robot handle to the start location, located at the origin of the coordinate system (0,0). Throughout the trial, the eyes remained fixated at the target location (0,20). Following the go cue, the participants moved the handle mostly in the vertical direction from the start to the target location.

The *x* and *y* positions of the robot handle were recorded to analyze hand movements. The raw hand position signal was filtered with a third-order, zero-phase lag, 10-Hz Butterworth filter. We used a combined velocity and acceleration criterion to find movement onsets and offsets. Movement onsets were defined as the moment in time when hand velocity exceeded 5% of peak velocity and the hand accelerated at a rate of 5% of peak acceleration. Movement offsets were defined as the moment in time that hand velocity fell below 5% of peak velocity and the hand decelerated at a rate of 5% of peak acceleration. Hand movement duration was defined as the difference between on and offset.

The *x* and *y* positions of gaze were recorded relative to the vertical monitor. The raw eye position data were upsampled to match the 1000-Hz time frame of the hand movement data, and eye data were filtered (second-order, zero-phase lag, low-pass, 15-Hz cut-off frequency Butterworth filter). Microsaccade onsets and offsets were identified using a velocity-based algorithm (Engbert & Mergenthaler, 2006). We first defined a two-dimensional (2D) velocity space based on the horizontal and vertical velocities. Then, we computed separate thresholds for vertical and horizontal velocities to define an elliptic threshold in the 2D velocity space. These thresholds were computed in units of median-based standard deviations (*SD*) for each direction. To improve the signal-to-noise ratio, it is recommended to use a relative velocity threshold (λ). We set λ equal to 5 multiples of the median-based SD estimation, as this value represents an optimum signal-to-noise ratio (Engbert & Mergenthaler, 2006). Additionally, microsaccades had to have a minimum duration of 40 data points (i.e., 40 ms). Microsaccade onset was defined as the first velocity value that moved outside the elliptic threshold, whereas the microsaccade offset was determined as the last velocity value outside the elliptic threshold. We calculated the microsaccade duration as the time interval between microsaccade onset and offset, and the microsaccade amplitude as the Euclidean distance between microsaccade onset and offset. An example of two detected microsaccades in a single reach-visible trial is shown in Figure 2. Note that microsaccades were only detected between the time at which the target appeared (show target) and the trial end, which coincided with the end of the movement in some conditions.

To determine the microsaccadic rate, we generated (for each trial) a time-varying binary microsaccade state variable that was set to 1 while a microsaccade was occurring and was otherwise set to 0. To compute the time-varying microsaccade rate for each participant and condition, we first aligned all contributing trials to the go signal, summed the microsaccade state across these trials, and then divided by the number of trials. This signal represented the proportion of trials in which microsaccades occurred as a function of time. To convert this signal into an estimate of the microsaccade rate, we divided it by the average duration of a microsaccade (see Results section). This method for calculating microsaccade rate was adapted from a method of calculating saccade rate reported in Fooken and Spering (2020). For comparison, we also estimated microsaccade rates by counting the number of microsaccade onsets at each millisecond and then computed a weighted sum using the causal kernel (White & Rolfs, 2016). The two methods yielded statistically the same results.

For each participant and condition, we evaluated the average microsaccade rate over three time zones: (1) The *initial fixation zone* was defined as the time between 1.75 and 1.65 seconds before the go signal. Note that, because the fixation duration before the cue was at least 1.8 seconds, this time window ensured that, in all trials, participants were already fixating the target. (2) The *pre-cue zone* was defined as the time between 0.25 second before the go signal and the time of the go signal. To ensure that our results were not sensitive to the selected time interval, we also calculated average microsaccade rates in pre-cue zones ranging from 0.375 second to the go signal and 0.5 second to the go signal. (3) The *post-inhibition zone* was defined as the time between 0.3 and 0.8 second following the go signal. (4) The *movement zone* was normalized within the cursor tracking, reach visible, and reach invisible conditions to account for differences in movement durations. For each trial, we counted the number of microsaccades that occurred during the condition-specific movement phase—that is, the time interval between hand movement onset and offset for reaching trials or the predetermined time interval for the cursor tracking and cue monitoring conditions. To obtain an average microsaccade rate, we summed the number of microsaccade onsets and the movement duration across all trials per participant and condition and then divided the total number of microsaccades by the total movement duration. Note that we did not include the cue monitoring condition in the analysis of the movement zone.

Finally, we defined two more zones to investigate the sharp microsaccade inhibition following the transient go cue. The pre-cue zone was defined as the time between 0.1 second before the go cue and the time of the go cue, and the post-cue zone was defined as the time between 0.15 and 0.25 second after the go signal.

### Data exclusion

We excluded trials in which participants violated task instructions based on the following criteria. First, we excluded trials in which the hand reaction time was below 100 ms or the eye data were missing (309 trials or 7.6%). For the remaining trials, we defined a time window starting from the time at which the target appeared (show target) to the time at which the cursor arrived at the target in the reaching conditions or the predetermined time interval for the cursor tracking and cue monitoring conditions. We excluded trials where target fixations started too late (≥100 ms after target appearance; 32 trials or 0.8%) or where target fixations ended too early (≥100 ms before the cursor was at the target; 183 trials or 4.5%). We also excluded trials in which the eye landed more than 6 cm away from the target (278 trials or 6.8%). The total number of excluded trials across participants was 581, or 14.2%. The remaining 3499 trials were used in the subsequent data analysis.

## Results

In this study, we investigated the modulation of microsaccades in different visuomotor coordination tasks. Participants viewed a stationary target for 1.8 to 2 seconds before seeing a go cue (color change). Following the go cue, participants continued viewing the target (cue monitoring), passively viewed the cursor move to the target (cursor tracking), or performed a reaching movement to the target with (reach visible) or without (reach invisible) cursor feedback. While maintaining gaze at the target, participants naturally elicited microsaccades; however, task demands shaped whether and when microsaccades occurred.

### Visuomotor behavior and microsaccade properties

**Figure 3.**
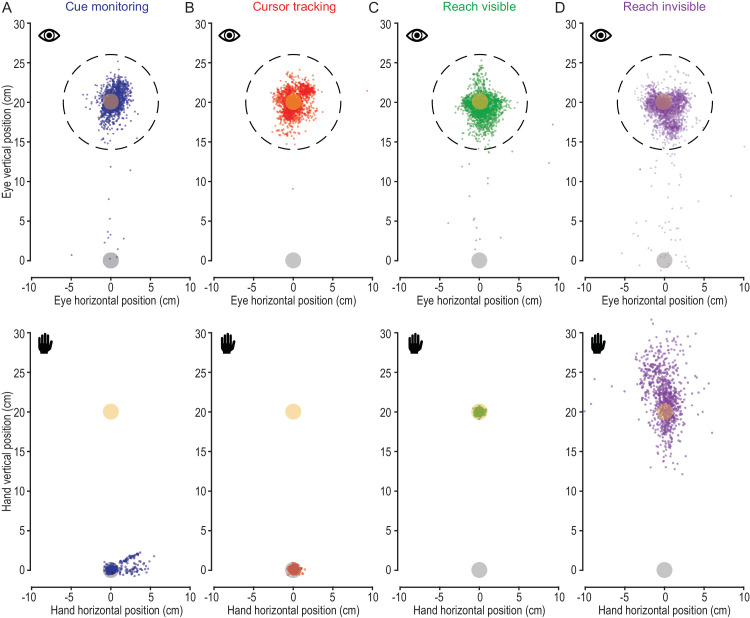
Eye (top) and hand (bottom) positions in different task conditions. In all conditions, participants fixated the target throughout the trial. Eye positions following saccades are represented as individual dots in the top panels. Trials in which saccades landed more than 6 cm away from the target (dashed circles) were excluded from analysis (see Methods). The final hand positions are indicated as individual dots in the lower panels. (**A**) In the cue monitoring condition, both the hand and the cursor remained at the start location. (**B**) In the cursor tracking condition, the hand remained at the start location while the cursor moved to the target. (**C**) In the reach visible condition, both the hand and the cursor moved from the start to the target location. (**D**) In the hand invisible condition, the hand moved from the start to the target location while no cursor movement was visible.

Figure 3 illustrates an overview of eye and hand position in the different task conditions. In the top panels of Figure 3, the 2D gaze position following each saccade that occurred across a trial is illustrated by a single dot. Saccades that landed farther than 6 cm away from the target were excluded from analysis (see Methods). In the bottom panels of Figure 3, the hand position at the end of the movement sequence is shown for each task condition. Here, the difference between the conditions becomes apparent. Whereas the hand remained at the start location in the cue monitoring and cursor tracking condition (Figures 3A and 3B), participants moved their hands to the target location in the reach visible and invisible condition (Figures 3C and 3D).

To assess whether microsaccade parameters were affected by different task conditions, we conducted a repeated-measure one-way ANOVA. We found no effect of task condition on microsaccade duration, *F*(3, 48) = 2.2, *p* = 0.095; amplitude, *F*(3, 48) = 1.1, *p* = 0.36; or peak velocity, *F*(3, 48) = 0.6, *p* = 0.63. We found that, across conditions, microsaccades were on average 86 ms (*SE* = 4 ms) in duration and 0.95 cm (*SE* = 0.03 cm) in amplitude, and they had a peak velocity of 23.9 cm/s (*SE* = 0.9 cm/s).

To assess whether hand movements were affected by cursor feedback (reach visible vs. reach invisible), we conducted a paired *t*-test. We found that hand reaction time increased, *t*(16) = 3.8, *p* = 0.001, in the reach invisible condition (*M* = 0.45, *SE* = 0.02 second) compared with the reach visible condition (*M* = 0.38, *SE* = 0.01 second). Whether cursor feedback was given did not affect hand movement duration, *t*(16) = 1.7, *p* = 0.10, or hand peak velocity, *t*(16) = 1.6, *p* = 0.129. Participants moved their hand with a mean duration of 1.36 seconds (*SE* = 0.04 second) and a mean hand peak velocity of 29.7 cm/s (*SE* = 1.08 cm/s). These findings indicate that participants were slower to start the hand movement when they could not see the cursor, but they reached the target relatively similarly.

### Microsaccades are reduced in anticipation of task-relevant events

**Figure 4.**
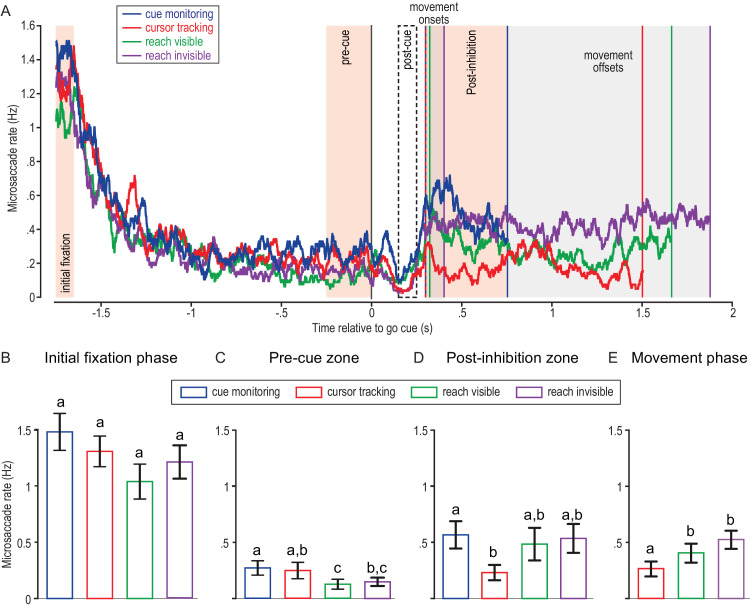
Microsaccade rates as a function of the visuomotor task. (**A**) Microsaccade rates, combining all trials from all participants, relative to the time of the go cue. Different task conditions are indicated by color. Zones used for statistical analysis are indicated by shaded areas. Note that the movement zone was normalized by the different movement durations in each condition (see Methods). (**B–E**) Post hoc comparison of different task conditions in the initial fixation zone (**B**), pre-cue zone (**C**), post-inhibition zone (**D**), and movement zone (**E**). Letters indicate which conditions were not different from each other in post hoc analyses. Bars with the same letter indicate no significant statistical difference, whereas bars with different letters indicate a significant difference between the corresponding conditions.

Figure 4A illustrates how the microsaccade rate changed as a function of the unfolding task. At the start of the target fixation period, the microsaccade rate was between 1 and 1.5 Hz before gradually decreasing until the time of the go cue. After the go cue, the microsaccade rate remained reduced (compared with the time of initial fixation) but increased slightly compared with the time before the cue. To quantitatively assess the change in microsaccade rate, we conducted a 2 × 4 repeated-measures analysis of variance (ANOVA).

Irrespective of the duration of the pre-cue zone (250, 375, or 500 ms), we found a main effect of zones, *F*(1, 16) ≥ 84.6, *p* <.001, and task conditions, *F*(3, 48) ≥ 3.7, *p* = 0.018, but no significant interaction between zones and task conditions, *F*(3, 48) ≤ 1.1, *p* = 0.35. For post hoc analysis, we decided to use the 250-ms pre-cue zone duration, as this time interval was most closely matched to the initial fixation window. However, we found the same statistical effects for all pre-cue zone durations. Post hoc analysis of the effect of zones showed that the microsaccade rate was greater in the initial fixation zone (*M* = 1.26, *SE* = 0.11 Hz) than in the pre-cue zone (*M* = 0.20, *SE* = 0.05 Hz; *p* < 0.001). For the effect of task conditions, the post hoc analysis showed that the microsaccade rate was larger for the cue monitoring (*M* = 0.88, *SE* = 0.10 Hz) than for the reach visible condition (*M* = 0.58, *SE* = 0.08 Hz; *p* = 0.046).

To further explore the effect of task conditions, we ran separate one-way ANOVAs to assess the difference between the four conditions in each zone. In Figures 4B to 4E we use letters to indicate statistical differences in the post hoc analysis. In each panel, two bars that share a letter are not statistically different. In the initial fixation zone (Figure 4B), we did not find an effect of task condition, *F*(3, 48) = 2.4, *p* = 0.075. In the pre-cue zone (Figure 4C), there was a main effect of task condition, *F*(3, 48) = 4.1, *p* = 0.011. Post hoc analysis revealed that the reach visible condition had a greater reduction in microsaccade rate than the cue monitoring condition (*p* = 0.014) and the cursor tracking condition (*p* = 0.048). The reach invisible condition also had a greater reduction but, in this case, compared with cue monitoring only (*p* = 0.048). Thus, the reduction in microsaccade rate was even greater in anticipation of the go cue in the reaching conditions compared with the non-movement conditions.

In the movement zone, the analysis was split into two separate time windows because in the cue monitoring condition the movement phases ended right after the post-inhibition rebound (Figure 4A). The first analysis included all conditions, but the interval considered for analysis was limited to the duration of the cue monitoring condition. The one-way ANOVA exhibited a main effect of task conditions, *F*(3, 48) = 3.0, *p* = 0.041. The microsaccade rate was smaller for the cursor tracking (*p* = 0.019) than for the cue monitoring condition (Figure 4D). The second analysis compared the cursor tracking, reach visible, and reach invisible conditions, including the whole movement duration zone. We found a main effect of task conditions, *F*(2, 32) = 7.2, *p* = 0.003. The microsaccade rate was smaller for the cursor tracking condition than for the reach invisible condition (*p* = 0.017) and reach visible condition (*p* = 0.040) (Figure 4E).

### Microsaccade inhibition following the go cue

Previous studies have reported an inhibition phase between 150 and 200 ms after the presentation of a sudden visual stimulus (Engbert & Kliegl, 2003; Hafed & Krauzlis, 2010; Rolfs, 2009; Rolfs, Kliegl, & Engbert, 2008). To assess whether a similar inhibition phase was observed in our task, we compared microsaccade rates between the pre- and post-cue zones using a 2 × 4 repeated-measures ANOVA. We found only a main effect of zones, *F*(1,16) = 8.5, *p* = 0.010. Overall, the microsaccade rate in the pre-cue zone (*M* = 0.21, *SE* = 0.05 Hz) was greater than that in the post-cue zone (*M* = 0.11, *SE* = 0.02 Hz; *p* = 0.010).

## Discussion

In this study, we investigated how microsaccade modulation relates to sensorimotor control in goal-directed reaching. Participants fixated a visual target and were cued to actively reach to the target, passively view a cursor move to the target, or wait for the trial end. In support of our first hypothesis, we found that, in all conditions, microsaccade rates, which were initially high during target fixation, gradually decreased in anticipation of the go cue. The reduction in microsaccade rate in anticipation of the go cue was greatest in the active movement conditions, suggesting that action preparation was associated with higher attentional load. During the movement phase, microsaccades were most strongly reduced when passively viewing the cursor move to the target (tracking condition), but a reduction in microsaccade rates was observed in all conditions. This universal reduction may be linked to the anticipation of sensory events associated with movement termination. These may include the removal of the visual target (all conditions), visual and proprioceptive events associated with the arrival of the hand at the target (reach conditions), and the arrival of the viewed cursor at the target (reach visible and cursor tracking conditions). These results show that sensorimotor control processes, including the prediction of sensory events, that take place during movement do not prevent microsaccade modulation. Overall, our findings suggest that the reduction in microsaccade rate is strongly linked to the anticipation of relevant sensory events but is neither enhanced nor strongly dampened during goal-directed reaching.

### Reduction in microsaccade rate is strongest for visual motion

We evaluated several hypotheses about how microsaccade rate would change during our task (Figure 1E). We found support for our first hypothesis that microsaccade rates during target fixation would gradually decrease in anticipation of the go cue in all task conditions. Specifically, microsaccade rates were systematically reduced starting about 1 second before the go cue. These findings are in line with previous work highlighting that microsaccades are indicative of observers’ temporal expectation of external events (Abeles et al., 2020; Amit et al., 2019; Badde et al., 2020; Dankner, Shalev, Carrasco, & Yuval-Greenberg, 2017; Palmieri, Fernández, & Carrasco, 2023).

Following the go cue, we found that task conditions differentially affected microsaccade modulation. A reduction in microsaccade rate was observed in both movement conditions but also in the two control conditions (i.e., the cursor tracking and cue monitoring conditions), indicating that the reduction in microsaccade rate during movement may not be related to sensorimotor control mechanisms, per se. Note that, in the cue monitoring condition, the event that occurred after the go cue was removal of the target, marking the end of the trial. The fact that the microsaccade rate was reduced in this condition suggests that modulation is linked to anticipating both the presentation of a sensory cue and the removal of a sensory cue. More generally, saccadic modulation may be linked to any sensory event that is linked to changing task demands.

The reduction in microsaccade rate was greatest when participants passively viewed the cursor move from the start location to the target (cursor tracking). This finding could indicate that seeing the cursor motion with peripheral vision was linked to a strong anticipation of the moment that the cursor contacted the foveated target while controlling fixational stability without eliciting microsaccades—that is, by slow control (Epelboim & Kowler, 1993; Kowler & Steinman, 1979). In line with this idea, previous research has found that fixation position is biased in the direction of a peripherally moving target (Makin, Poliakoff, Ackerley, & El-Deredy, 2012). Another explanation for the observed microsaccade modulation is that participants may have suppressed microsaccades to increase visual motion processing, as motion sensitivity is altered during saccades (Binda & Morrone, 2018; Burr, Holt, Johnstone, & Ross, 1982; Castet & Masson, 2000; Shioiri & Cavanagh, 1989). Such mechanisms might be similar to the suppression of saccades during manual interception. Here, catch-up saccades that realign the eyes with the moving target are suppressed in anticipation of the time of interception (Fooken, Kreyenmeier, & Spering, 2021; Goettker, Brenner, Gegenfurtner, & de la Malla, 2019; Mrotek & Soechting, 2007; Schroeger, Goettker, Braun, & Gegenfurtner, 2024). This idea is also in line with our observation that participants elicited fewer microsaccades during the movement phase as compared with the initial fixation phase, suggesting the importance of uninterrupted visual input during ongoing sensorimotor control.

### Microsaccades as indicators of attentional processing

Whereas we did not find that the reduction in microsaccade rate was greater in the movement phase in conditions that required an active movement, we did find that, in these conditions, microsaccades prior to the go cue were more strongly suppressed than in the conditions that did not require movement. These results suggest that the consequences of the sensory event affect how much microsaccades are reduced in anticipation of a predictable event. The greater reduction in microsaccade rate observed when an active movement was required after the go cue suggests that these conditions were associated with a higher attentional load. Previous work has found that microsaccades are more strongly modulated in tasks that require a high perceptual load (Pastukhov & Braun, 2010; Xue et al., 2017) or working memory load (Dalmaso, Castelli, Scatturin, & Galfano, 2017). When perceptual tasks require a manual response, the reduction in microsaccade rate is greater when manual responses are elicited more quickly (Betta & Turatto, 2006; Loughnane, Newman, Tamang, Kelly, & O'Connell, 2018). Our findings suggest that having to prepare a goal-directed reaching movement increased participants’ attentional load.

This interpretation aligns with previous findings showing that visual attention is tightly coupled to the preparation of goal-directed manual actions. Specifically, attention is selectively allocated to movement-relevant locations even before movement onset, highlighting the role of attention when planning prehension movements (Schiegg, Deubel, & Schneider, 2003). Preparing for a goal-directed hand movement has been shown to involve a selective allocation of visual attention toward action-relevant locations, a process termed “visual preparation” (Baldauf & Deubel, 2010). In the present study, participants always reached to the same position that was already fixated at the start of reach. We observed a slight increase in large saccades toward the hand in the two movement conditions (see Figures 3C and 3D), suggesting that visual attention was to some degree divided between the reach start and the reach goal. Future studies could investigate the effect of visual spatial attention by decoupling the reaching target from the fixation target. The current findings more generally support the idea that preparing to interact with a visual target through reaching strongly engages visuospatial attention.

A task-dependent modulation in oculomotor control has been associated not only with attentional load per se but also more generally with varying levels of arousal (Burlingham, Mirbagheri, & Heeger, 2022; Contadini-Wright, Magami, Mehta, & Chait, 2023; Wang & Munoz, 2021). Arousal-dependent modulation of microsaccades in anticipation of a sensory event could facilitate the synchronization of internal neural states and external sensory events (Buonocore & Hafed, 2023). The observation that the reduction in microsaccade rate was greatest when a hand movement followed the go cue could indicate that the synchronization between internal and external events is especially relevant when preparing to interact with the environment.

### Top–down versus bottom–up control of microsaccades

Microsaccades are functionally important for both visual and oculomotor control (Collewijn & Kowler, 2008; Hafed, Chen, & Tian, 2015; Martinez-Conde, Macknik, & Hubel, 2004; Rolfs, 2009). Generally, microsaccades are important to prevent perceptual fading (Martinez-Conde, Macknik, Troncoso, & Dyar, 2006; McCamy et al., 2012) and to increase visual acuity in visual discrimination tasks (Ko, Poletti, & Rucci, 2010; Rucci, Iovin, Poletti, & Santini, 2007) and in high-precision action tasks (Valsecchi & Gegenfurtner, 2015; Winterson & Collewijn, 1976). Although many studies have highlighted that microsaccades are subject to voluntary control (Steinman, Cunitz, Timberlake, & Herman, 1967; Willeke et al., 2019), the frequency and direction of microsaccades are also modulated by visual factors (Cui, Wilke, Logothetis, Leopold, & Liang, 2009; Hicheur, Zozor, Campagne, & Chauvin, 2013). When salient changes in the visual scene occur, such as a full-screen flash or the disappearance of a stimulus, microsaccades are inhibited following the visual event (Buonocore et al., 2017; Engbert & Kliegl, 2003; Rolfs et al., 2008; Tian, Yoshida, & Hafed, 2016), a mechanism that has been proposed to synchronize external visual events with internal brain states (Buonocore & Hafed, 2023). Consistent with these results, we observed a distinct dip in the microsaccade rate approximately 150 to 200 ms after the visual go cue, irrespective of task condition.

Although we did not find a difference between microsaccade rates across conditions in the post-cue window, we cannot rule out that top–down mechanisms further modulate microsaccade inhibition. Previous work has shown that the magnitude and duration of saccadic inhibition—following a visual event—are shaped by attentional task demands in both free viewing (Glaholt & Reingold, 2018; Reingold & Stampe, 2004) and in visually guided saccades (Buonocore & McIntosh, 2013). Moreover, the generation and inhibition of microsaccades have been linked to neural processes of evidence accumulation and decision formation (Loughnane et al., 2018). In our task, microsaccade rates were already very low at the time of the visual go cue. Thus, it is possible that we were not able to detect additional top–down influences on microsaccade inhibition. Taken together, our results highlight that the time course and magnitude of microsaccade modulation reflect both bottom–up responses to salient sensory events as well as top–down cognitive processes.

### Interdependence of sensory and oculomotor processes

Behaviorally, our results resemble previous findings that show that microsaccades are gradually reduced in anticipation of sensory events. Such a modulation has been termed oculomotor inhibition or freezing (Abeles et al., 2020; Amit et al., 2019; Badde et al., 2020; Dankner et al., 2017) and—in the case of catch-up saccades in manual interception tasks—saccadic suppression (Fooken et al., 2021; Goettker et al., 2019; Mrotek & Soechting, 2007; Schroeger et al., 2024). However, more traditionally, the term oculomotor or (micro)saccadic inhibition or freezing has been used to refer to the sharp drop in microsaccade rate following a salient sensory transient (Buonocore & McIntosh, 2013; Reingold & Stampe, 2004; Rolfs et al., 2008; White & Rolfs, 2016). Neuronally, the inhibition of microsaccade generation in anticipation of, as opposed to following, a sensory event may be subject to separate neural control mechanisms (Buonocore & Hafed, 2023; Nobre & van Ede, 2018). Whether and how these processes are interdependent within and across individuals remain to be seen.

## Conclusions

In this study, we examined how microsaccade modulation relates to perceptual and sensorimotor control in goal-directed reaching. We found that the rate of microsaccades was consistently reduced in anticipation of a go cue that followed different task demands. The reduction in microsaccade rate immediately before the go cue was greatest in conditions that required an active hand movement, suggesting that movement preparation added attentional load to the sensorimotor system. Following the go cue, microsaccade rates remained reduced in all conditions, indicating that microsaccade rates are reduced not only in anticipation of external sensory events but more generally in anticipation of task-relevant events, such as the trial end (cue monitoring), the time the visual cursor contacted the target (cursor tracking), or the reach end (reach visible, reach invisible). Overall, the study suggests that microsaccade modulation is closely related to anticipating sensory events but does not necessarily link to ongoing self-generated hand movement control.

### Citation diversity statement

Recent work in several fields of science has identified a bias in citation practices such that papers from women and other minority scholars are under-cited relative to the number of such papers in the field (Bertolero et al., 2020; Caplar, Tacchella, & Birrer, 2017; Chatterjee & Werner, 2021; Dion, Sumner, & Mitchell, 2018; Dworkin et al., 2020; Fulvio, Akinnola, & Postle, 2021; Maliniak, Powers, & Walter, 2013; Mitchell, Lange, & Brus, 2013; Wang et al., 2021). Here, we sought to proactively consider choosing references that reflect the diversity of the field in terms of thought, form of contribution, gender, race, ethnicity, and other factors. First, we obtained the predicted gender of the first and last author of each reference by using databases that store the probability of a first name being carried by a woman (Dworkin et al., 2020; Zhou et al., 2020). By this measure (and excluding self-citations to the first and last authors of our current paper), our references contain 15.85% women (first)/women (last), 16.3% men/women, 18.29% women/men, and 49.55% men/men. This method is limited in that (a) names, pronouns, and social media profiles used to construct the databases may not, in every case, be indicative of gender identity; and (b) it cannot account for intersex, non-binary, or transgender people. Second, we obtained the predicted racial/ethnic category of the first and last author of each reference from databases that store the probability of a first and last name being carried by an author of color (Ambekar, Ward, Mohammed, Male, & Skiena, 2009; Chintalapati, Laohaprapanon, & Sood, 2023). By this measure (and excluding self-citations), our references contain 9.6% authors of color (first)/authors of color(last), 15.14% white authors/authors of color, 18.01% authors of color/white authors, and 57.25% white authors/white authors. This method is limited in that (a) names and Florida Voter Data to make the predictions may not be indicative of racial/ethnic identity, and (b) it cannot account for Indigenous and mixed-race authors, or those who may face differential biases due to the ambiguous racialization or ethnicization of their names. We look forward to future work that could help us to better understand how to support equitable practices in science.
